# Analytical validation and clinical evaluation of a high-sensitivity cardiac troponin I immunoassay for use in cats

**DOI:** 10.1371/journal.pone.0346522

**Published:** 2026-04-08

**Authors:** Jonathan A. Lidbury, Rachel C. Meyers, Ashley B. Saunders, Jordan P. Vitt, Sonya G. Gordon, Sukjung Lim, Ryan C. Fries, Jan S. Suchodolski, Joerg M. Steiner, Sonya Wesselowski

**Affiliations:** 1 Department of Small Animal Clinical Sciences, College of Veterinary Medicine and Biomedical Sciences, Texas A&M University, College Station, Texas, United States of America; 2 Department of Veterinary Clinical Medicine, University of Illinois, Urbana-Champaign, Illinois, United States of America; SRM University AP SEAS: SRM University AP School of Engineering and Sciences, INDIA

## Abstract

Cardiac troponin I (cTnI) is a cardiac specific biomarker of myocardial damage in humans, dogs, and cats. The ADVIA Centaur XP High-Sensitivity Troponin I assay (AC-cTnI-HS) has been validated for use in humans and dogs, but not for use in cats. The study objective was to analytically validate the AC-cTnI-HS assay for use in cats and to evaluate cTnI measurements in healthy cats compared to those with cardiac disease to assess the clinical utility of this assay. Surplus serum samples from cats were used for analytical validation. Intra- and inter-assay variability, dilutional parallelism, and spiking recovery were assessed. Serum samples from 106 client-owned cats were evaluated. This group was comprised of 51 clinically healthy cats (structurally normal echocardiogram, normal systemic blood pressure, and unremarkable complete blood count and biochemistry profile), 25 cats with stage B1 hypertrophic cardiomyopathy, 7 with stage B2 hypertrophic cardiomyopathy, 7 with stage C cardiomyopathy of any type, 8 with congenital heart disease, and 8 cats with transient myocardial thickening and/or suspected to have myocarditis. Inter-assay and intra-assay coefficients of variation were between 2.7–8.3% and 1.5–4.0%, respectively. The mean ± standard deviation observed to expected ratios for dilutional parallelism and spiking recovery were 124.3 ± 42.8% and 92.9 ± 6.2%, respectively. Healthy cats had significantly lower cTnI concentrations than cats with hypertrophic cardiomyopathy stage B1 (P = 0.012), stage B2 (P = 0.004), or any cardiomyopathy ACVIM stage C (P = 0.002). The AC-cTnI-HS assay is precise, reproducible, linear, and accurate for measurement of cTnI concentrations in serum from cats. This study confirms that measurement of serum cTnI holds promise to have clinical utility as it was able to detect differences in serum cTnI concentrations between healthy cats and those with cardiac disease.

## Introduction

Cardiac troponin I (cTnI) is a sensitive and specific cardiac biomarker, which is released quickly and persistently from cardiomyocytes into the blood stream in the face of myocardial damage [1]. Increased serum cTnI concentrations have been reported in cats with primary myocardial disease [2–11], as well as in cats with a variety of other abnormalities including transient myocardial thickening (TMT) [12–16], anemia [17], hyperthyroidism [18,19], renal disease [20–22], systemic hypertension [22], systemic inflammatory response syndrome [23], various critical illnesses [24], arrhythmias [25], and/or secondary to anesthetic events or drugs [26,27]. In cats with hypertrophic cardiomyopathy (HCM), multiple studies have shown that cTnI concentrations increase as disease severity increases [2,6–9], with two studies also demonstrating negative prognostic significance associated with elevated cTnI concentrations in cats with HCM [4,5].

Until recently, the ultrasensitive ADVIA Centaur TnI-Ultra assay was commonly utilized for the measurement of cTnI in dogs and cats, having been validated for use in both species [28,29]. This assay has since been replaced by the ADVIA Centaur XP High-Sensitivity Troponin I (AC-cTnI-HS) assay, which has superior diagnostic utility in humans compared to the previous generation assay [30] and improved analytical performance [31]. The specific qualifications that must be met for an assay to be termed “highly sensitive” include detection of cTnI precisely (with a coefficient of variation (CV) <10%) at or below the 99^th^ percentile upper reference limit in at least half the population [32]. The AC-cTnI-HS assay meets these criteria in humans, with a defined limit of quantification between 2.5–25,000 pg/mL (ng/L) [31]. The AC-cTnI-HS assay has also been validated for use in dogs, however the lowest cTnI concentration with a repeatable intra- and inter-assay CV < 10% in that species was > 20 pg/mL, suggesting worse analytical performance in dogs than in humans [33].

The study objective of this study was to analytically validate the AC-cTnI-HS assay for use in cats and to evaluate cTnI measurements in healthy cats compared to those with cardiac disease to assess the clinical utility of this assay in cats.

## Materials and methods

### Analytical validation

Left-over serum from diagnostic samples submitted to the Gastrointestinal Laboratory at Texas A&M University were pooled to create samples with AC-cTnI-HS concentrations of approximately 10, 20, 50, 100, 250, and 1,000 pg/mL. Intra-assay variability was assessed by measuring aliquots of these six serum pools ten times in the same assay run. Inter-assay variability was determined by measuring aliquots from the same six serum pools over ten consecutive days. The mean AC-cTnI-HS concentrations for the pools were 8, 21, 46, 96, 240, and 1,044 pg/mL. Aliquots of the three pools with the highest cTnI concentrations (96, 240, and 1,044 pg/mL) were serially diluted (1:2, 1:4, 1:8, and 1:16) with manufacturer provided sample diluent. Observed to expected (O/E) ratios were determined from these results. Spiking recovery accuracy was determined by mixing equal volumes of all six pools in all possible combinations (A + B, A + C, A + D etc.), with O/E ratios calculated from these results.

### Clinical evaluation

Cats were recruited for the clinical evaluation portion of the study from Texas A&M University Veterinary Medical Teaching Hospital faculty, student, and staff-owned cats as well as client-owned cats presenting to the cardiology service at the Veterinary Medical Teaching Hospital of Texas A&M University or the University of Illinois at Urbana-Champaign. Informed written consent was obtained from the owners of prospectively enrolled cats of studies that had been approved by the Institutional Animal Care and Use Committee of each university (Texas A&M University: IACUC 2023−0155 CA and IACUC 2020−0193 CA; University of Illinois: IACUC 19258). A small number of cats with cardiac disease evaluated by the cardiology service at Texas A&M University that had a cTnI concentration assessed by the AC-cTnI-HS assay as part of their diagnostic evaluation within the year prior to the start of the study were also identified and retrospectively enrolled.

Enrolled cats were allowed to receive gabapentin or trazodone the night prior to their evaluation and the morning of their evaluation at the owner’s discretion if these medications had been previously prescribed by a veterinarian to decrease the stress elicited by veterinary visits. Intravenous or intramuscular sedation with butorphanol was also allowed, as needed, based on the cat’s cooperation and demeanor once in the hospital. Serum samples were obtained via venipuncture of the external jugular or medial saphenous vein from all cats at the time of enrollment at both institutions. These samples were placed into plain serum tubes, allowed to clot for at least 15 minutes, then centrifuged at 20℃ at 14,800 rpm for 10 minutes. After centrifugation, serum was separated from the packed cells, divided into aliquots, and frozen at −80° C, except for cats in which it was clinically indicated to assess cTnI concentration as part of the cat’s diagnostic assessment. In these instances, serum samples were submitted for immediate AC-cTnI-HS analysis. Serum samples from the University of Illinois were transferred from a −80° C freezer and shipped to Texas A&M University overnight on dry ice, with all samples confirmed to be frozen upon receipt. These samples, in addition to the stored samples at Texas A&M University, were thawed and analyzed in a single batch at the conclusion of study enrollment. Cats were assigned to the healthy group if they had no clinically important findings on history and physical examination, an average systolic Doppler blood pressure reading < 160 mmHg, a structurally normal echocardiogram, and an unremarkable complete blood count and biochemistry profile. Cats with cardiac disease were grouped based on their echocardiogram results and other relevant diagnostic testing at the time of their assessment, with cardiomyopathy diagnoses classified based on the 2020 American College of Veterinary Internal Medicine (ACVIM) consensus guidelines on the classification and diagnosis of cardiomyopathies in cats [34]. Cats with cardiomyopathy were grouped into HCM stage B1, HCM stage B2, and any cardiomyopathy stage C (encompassing cats with active or compensated congestive heart failure due to any underlying type of cardiomyopathy). Cats diagnosed with one or more congenital cardiac abnormalities were assigned to the congenital group, while cats diagnosed with TMT or those suspected to have myocarditis based on cTnI concentrations presumed to be severely elevated were grouped together in a TMT/myocarditis suspect group.

### Statistical methods

Continuous variables were assessed for normality using Anderson-Darling tests and visual inspection of q-q plots. The data were not normally distributed and were therefore expressed as median (range). Comparisons of age and AC-cTnI-HS concentrations among groups of cats were made using Kruskal-Wallis tests, followed by a Dunn’s post-test with healthy cats as the reference group. As cTnI concentrations were used to select cases for the suspected myocarditis group, concentrations were not statistically compared between this group of cats and the others. The correlation between age and AC-cTnI-HS concentration in healthy cats was assessed using Spearman’s correlation coefficient. A preliminary reference interval was determined by calculating the central 95^th^ percentile of cTnI concentrations in healthy cats. Statistical significance was set at P < 0.05. A statistical software package was used for analysis (GraphPad Prism v.8, GraphPad, GraphPad Software, San Diego, CA).

## Results

### Analytical validation

**Fig 1:**
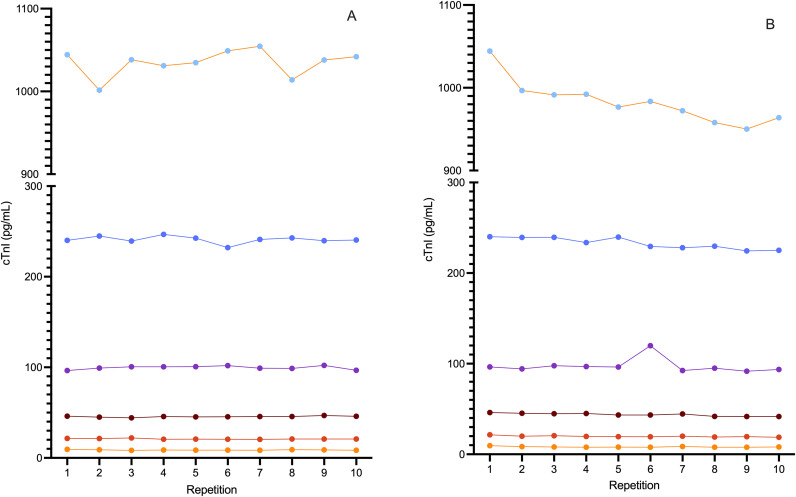
Inter and intra-assay variability of the ADVIA Centaur Cardiac Troponin I High Sensitivity assay using serum samples in cats presented in graphical format. Six serum pools of varying cTnI concentration were run repeatedly on the same assay run (intra-assay variability, panel A) and across different assay runs (inter-assay variability, panel B).

**Fig 2:**
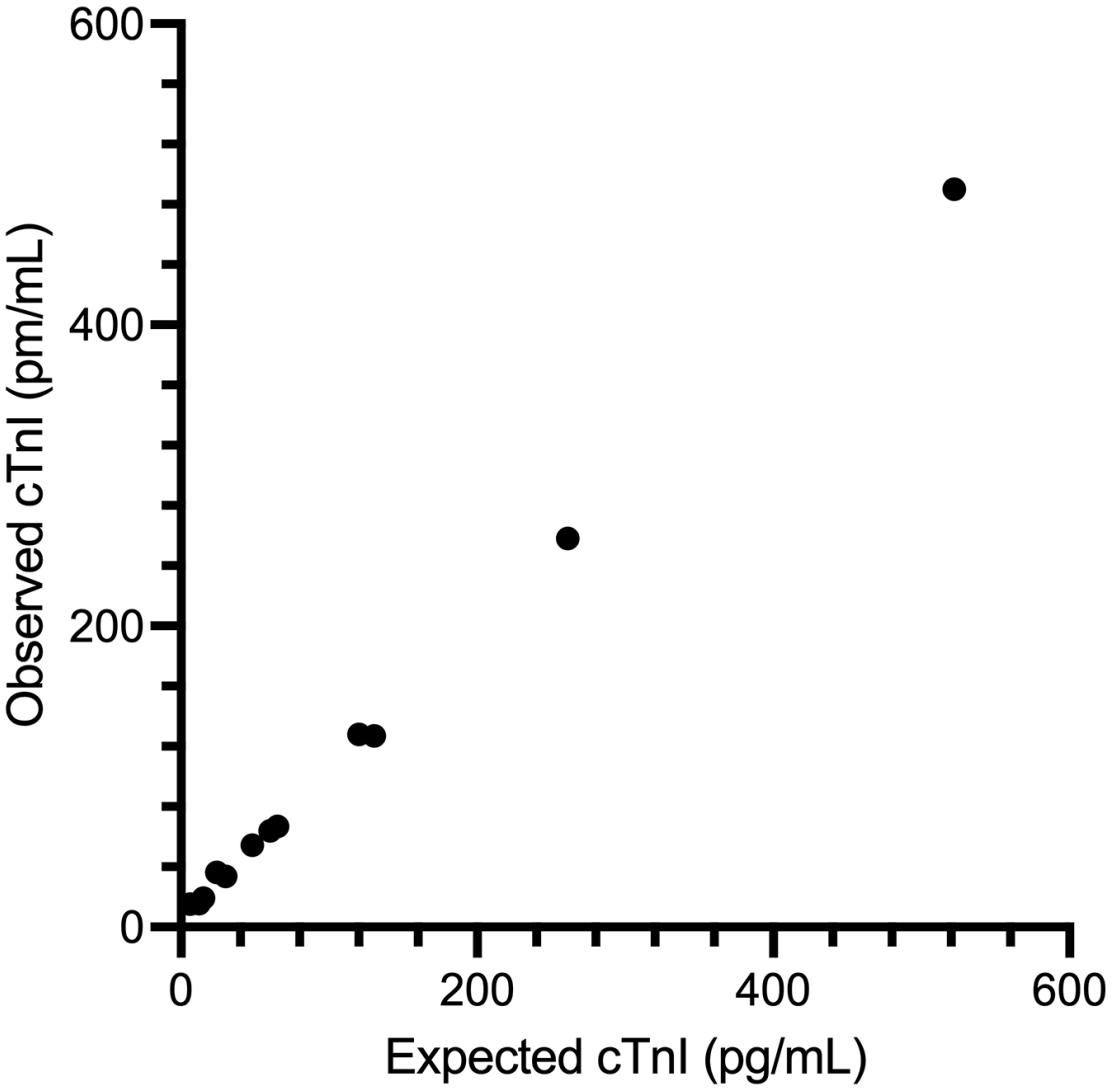
Dilutional parallelism results of the ADVIA Centaur Cardiac Troponin I High Sensitivity assay for measurement of cardiac troponin I in serum samples from cats in graphical format. Three serum pools of varying cTnI concentration were serially diluted (1:2, 1:4, 1:8, and 1:16) with manufacturer provided sample diluent. Observed values are plotted against expected values.

**Fig 3:**
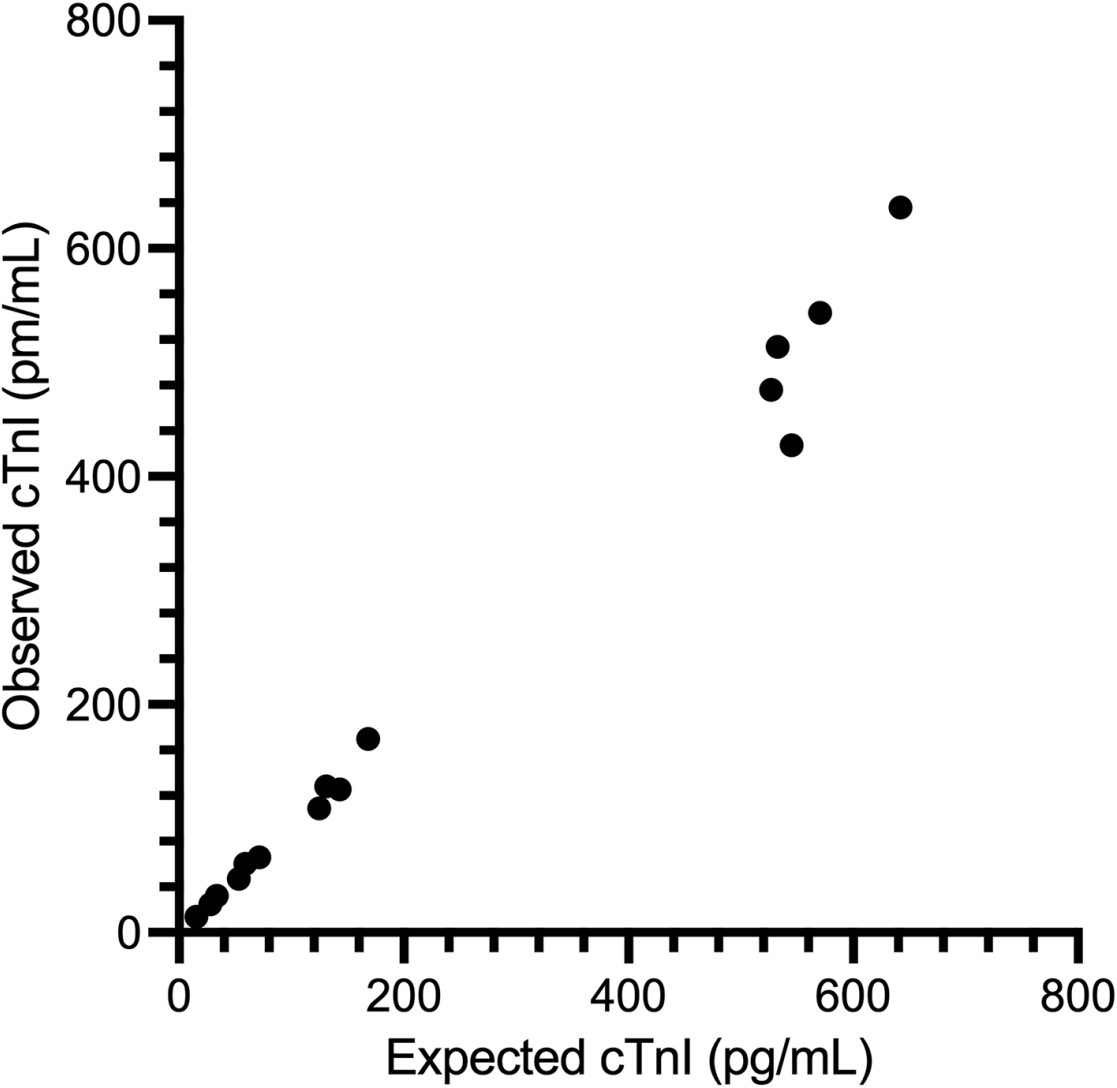
Spiking recovery results for the ADVIA Centaur Cardiac Troponin I High Sensitivity assay for measurement of cardiac troponin I in feline serum in graphical format. Equal volumes of six pools of varying cTnI concentration were mixed in every possibly combination. Observed values are plotted against expected values.

Intra-assay %CV were between 1.5–4.0%. Inter-assay coefficients of variation (%CV) were between 2.7–8.3%. (Table 1, Fig 1). The mean ± standard deviation O/E ratio for dilutional parallelism was 124.3 ± 42.8% with a range of 93.9–150.0% (Table 2, Fig 2). The mean ± standard deviation O/E ratio for spiking recovery was 92.9 ± 6.2% with a range of 78.3–101.7% (Table 3, Fig 3).

**Table 1:** Reproducibility and precision of the measurement of cardiac troponin I in serum samples from cats using the ADVIA Centaur Cardiac Troponin I High Sensitivity assay.

	Serum pool	Mean (pg/mL)	SD (pg/mL)	CV (%)
Intra-assay variability (n = 10)	1	9	0.3	4
	2	21	0.5	2.3
	3	46	0.7	1.5
	4	100	2	2
	5	241	3.9	1.6
	6	1035	16	1.5
Inter-assay variability (n = 10)	1	8	0.5	6.5
	2	20	0.8	3.8
	3	44	1.6	3.7
	4	97	8.1	8.3
	5	233	6.4	2.7
	6	983	26.5	2.7

CV: coefficient of variation; SD: standard deviation.

**Table 2:** Dilutional parallelism results of the ADVIA Centaur Cardiac Troponin I High Sensitivity assay for measurement of cardiac troponin I in serum samples from cats in tabular form.

Serum pool	Dilution	Observed cTnI concentration (pg/mL)	Expected cTnI concentration (pg/mL)	O/E ratio (%)
1	Undiluted	96		
	1:2	54	48	112.5
	1:4	36	24	150.0
	1:8	16	12	133.3
	1:16	15	6	250.0
2	Undiluted	240		
	1:2	128	120	106.7
	1:4	64	60	106.7
	1:8	34	30	113.3
	1:16	19	15	126.7
3	Undiluted	1044		
	1:2	490	522	93.9
	1:4	258	261	98.9
	1:8	127	131	96.9
	1:16	67	65	103.1

Assay linearity was assessed at dilutions of 1:2, 1:4, and 1:8. cTnI: cardiac troponin I; O:E observed to expected ratio.

**Table 3:** Spiking recovery results for the ADVIA Centaur Cardiac Troponin I High Sensitivity assay for measurement of cardiac troponin I in feline serum in tabular form.

Serum pool combination		Observed cTnI concentration (pg/mL)	Expected cTnI concentration (pg/mL)	O/E ratio (%)
A	+B	14	15	93.3
A	+C	25	28	89.3
A	+D	47	53	88.7
A	+E	109	125	87.2
A	+F	476	527	90.3
B	+C	32	34	94.1
B	+D	60	59	101.7
B	+E	128	131	97.7
B	+F	514	533	96.4
C	+D	66	71	93.0
C	+E	125	143	87.4
C	+F	427	545	78.3
D	+E	170	168	101.2
D	+F	544	570	95.4
E	+F	636	642	99.1

For spiking recovery experiments, each sample was spiked into each of the other samples. cTnI: cardiac troponin I; O:E observed to expected ratio.

### Clinical evaluation

**Fig 4:**
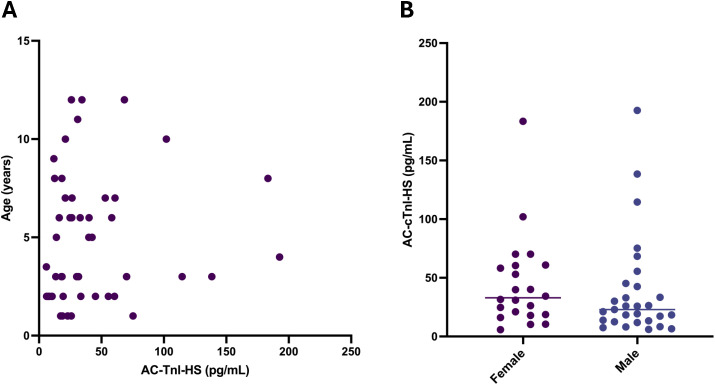
Effect of age and sex on cardiac troponin I concentrations in healthy cats. There was no correlation between age and cardiac troponin I concentrations in 51 healthy cats (r_s_ = 0.233, P = 0.100) with the ADVIA Centaur XP High-Sensitivity Troponin I assay (panel A). There was also no significant difference (P = 0.208) between cardiac troponin I concentrations in male and female healthy cats (panel B).

**Fig 5:**
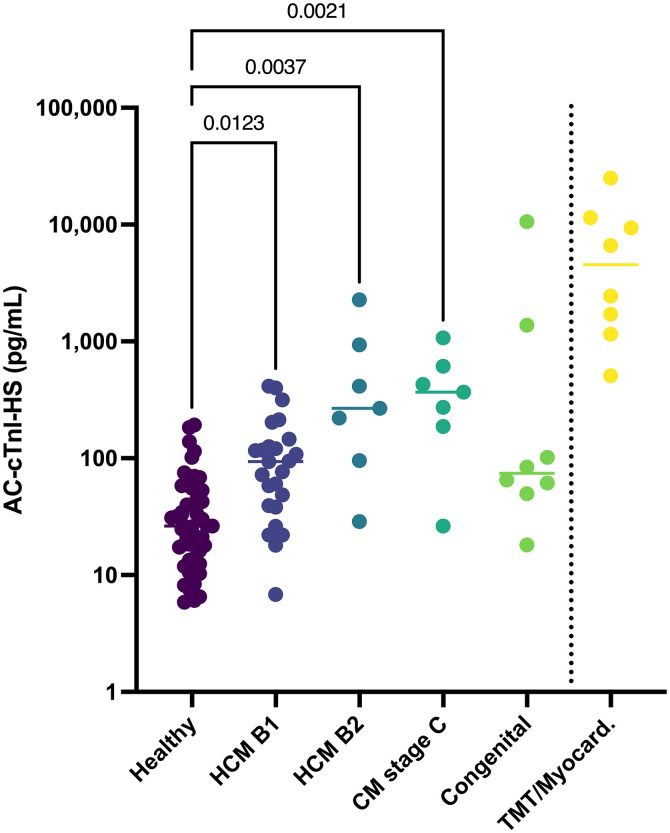
Cardiac troponin I concentrations in healthy cats and cats with various cardiac diseases. Cardiac troponin I concentrations measured with the ADVIA Centaur XP High-Sensitivity Troponin I assay in the serum of healthy cats and cats with hypertrophic cardiomyopathy (HCM) stage B1, HCM stage B2, any type of cardiomyopathy in stage C, congenital heart disease (congenital), or transient myocardial thickening and/or myocarditis suspects (TMT/myocard.) are depicted. * As cTnI concentrations were used to select cases for the suspected myocarditis group, concentrations were not statistically compared between this group of cats and the others.

Serum samples from 106 client-owned cats were analyzed (Table 4). The median age was 5 years (range: 0.4–15 years) and the median body weight was 5.1 kg (standard deviation: ± 1.3 kg). There were 40 female cats (9 intact) and 66 male cats (8 intact). The subgroup sizes were as follows: healthy cats (n = 51), HCM stage B1 (n = 25), HCM stage B2 (n = 7), any cardiomyopathy ACVIM stage C (n = 7), congenital heart disease (n = 8), or TMT and/or myocarditis suspect (n = 8). Five cats with congenital heart disease had a single defect (mitral valve dysplasia, atrial septal defect, ventricular septal defect, double outlet right atrium, and complete atrioventricular septal defect) and three had more than one congenital defect (ventricular septal defect and mitral valve dysplasia, partial atrioventricular septal defect with aortic valve dysplasia, and combined mitral and tricuspid valve dysplasia). All cats with congenital heart disease were considered to have mild disease except for the cat with a double outlet right atrium that had concurrent congestive heart failure and the cat with the complete atrioventricular septal defect that had concurrent congestive heart failure, pulmonary hypertension, and a left atrial thrombus. Amongst the groups, there was only a significant age difference between the cats with HCM stage B1 and the cats with congenital heart disease (P = 0.016). There was no significant correlation between age and AC-cTnI-HS concentrations in healthy cats (r_s_ = 0.233, P = 0.100) (Fig 4A). There was no significant difference between AC-cTnI-HS concentrations in healthy male cats versus female cats (P = 0.208) (Fig 4B). Cats with HCM stage B1 (P = 0.012), HCM stage B2 (P = 0.004), or any cardiomyopathy stage C (P = 0.002) had significantly higher AC-cTnI-HS concentrations than healthy cats (Fig 5). There was no significant difference in AC-cTnI-HS concentrations between cats with congenital heart disease and healthy cats.

**Table 4:** Demographic data and serum cardiac troponin I concentration in healthy cats and cats with cardiac disease.

Group	Number	Age (years)	Sex (M/F)	Median (range) AC-cTnI-HS (pg/mL)
Healthy	51	3.0 (1.0-12.0)	29/22	26 (<8-193)
HCM stage B1	25	7.0 (1.0-14.0)	16/9	94 (<8-415)
HCM stage B2	7	7.0 (4.0-9.0)	6/1	268 (29 –2,270)
Any cardiomyopathy stage C	7	3.0 (0.4-14.0)	6/1	369 (26 –1,073)
Congenital heart disease	8	1.5 (0.8-9.0)	4/4	75 (18 –10,602)
TMT/myocarditis suspect	8	6.5 (0.6-14.0)	5/3	4,532 (510−25,001)*

Demographic data and cardiac troponin I concentrations as measured by the ADVIA Centaur XP High-Sensitivity Troponin I (AC-cTnI-HS) assay are presented across clinical groups. Data for age and cardiac troponin I concentration are presented as median (range). HCM: hypertrophic cardiomyopathy; TMT: transient myocardial thickening. *As cTnI concentrations were used to select cases for the TMT/myocarditis suspect group, concentrations were not statistically compared between this group of cats and the others.

The 2.5th percentile and 97.5th percentile AC-cTnI-HS concentrations in healthy cats were 6.0 and 190 pg/mL, respectively. Thus, a preliminary reference interval for AC-cTnI-HS concentration in healthy cats based on these data in combination with the reproducibility data (which has a lowest mean pool concentration of 8.0 pg/mL) can reasonably be set at <8 pg/mL - 190 pg/mL.

## Discussion

This study indicates that the AC-cTnI-HS assay measures cTnI concentrations in the serum of healthy cats and cats with cardiac disease in a precise and reproducible fashion at all tested concentrations, including the lowest tested concentration of 8 pg/mL, with %CV < 10% for intra- and inter-assay variability. Across the study, 100% of the analyzed samples had measurable cTnI concentrations above the 2.5 pg/mL lower limit of detection established for humans for this assay, while 95.3% of cats had measurable cTnI concentrations at or above 8 pg/mL, the lowest concentration with proven precision and reproducibility based on the present data. Only five cats had cTnI concentrations measuring below 8 pg/mL. These findings suggest that the assay performance is superior in cats compared to dogs in this regard, as the lowest concentration where precise and reproducible concentrations can be measured in dogs has been reported as > 20 pg/mL [33].

The mean ± standard deviation O/E ratio for dilutional parallelism was 124.3 ± 42.8% with a range of 93.9–150.0%. This is outside typical target ranges of O/E between 80.0% and 120.0%. However, no samples with very high serum cTnI concentrations were used for this study. The sample with a neat serum cTnI concentration of 1044 pg/ml showed O/E ratios of 93.9, 98.9, 96.9, and 103.1%, all well within the range of 80–120%. Thus, the assay shows good reproducibility for samples in the middle or upper end of the working range and thus for the only samples that would possibly be diluted. Furthermore, this assay has a very wide working range (2.5–25,000 pg/mL in humans) and thus sample dilution of a patient sample will seldom be indicated.

The mean spiking recovery O/E ratio was 92.9 ± 6.2% with a range between 78.4 and 101.9%. This is within the typical target range of 80.0% to 120.0%, indicating the assay is analytically accurate (i.e., does not suffer from a matrix effect when measuring cTnI in cat serum). Ideally samples would have also been spiked with purified feline cardiac troponin. However, this reagent was not available.

Data derived from the clinical samples in this study showed that healthy cats had significantly lower AC-cTnI-HS concentrations than cats with any stage of cardiomyopathy. Differences in AC-cTnI-HS concentrations amongst groups of cats with various stages of cardiomyopathy were not detected. However, this may be reflective of the small sample sizes of cats with HCM stage B2 and cats in stage C secondary to any type of cardiomyopathy in this study, as previous studies have demonstrated a difference between cats with mild versus more severe stages of HCM [2,6–9]. The lack of difference in AC-cTnI-HS concentrations between healthy cats and cats with congenital heart disease is likely secondary to the considerable spectrum of severity of congenital cardiac disease along with the small sample size. Nevertheless, the assay was able to detect differences in cTnI between healthy cats and those with cardiac disease that would be expected to have varying degrees of myocardial injury, suggesting a potential for clinical utility.

In other species, such as dogs and humans, cTnI concentrations have been shown to increase with increasing age [29,33,35,36]. In the present data, age was not correlated with AC-cTnI-HS concentrations in cats. This aligns with several other feline studies that also failed to find a correlation between age and cTnI concentrations [2,7,8], although a single study did demonstrate a higher cTnI concentration in 13 cats over 10 years of age with no clinical evidence of cardiac disease compared to 20 control cats without cardiac disease [37]. Given that echocardiograms and screening for concurrent diseases was not pursued in the aged cats of that study [37], subclinical cardiac disease cannot be ruled out, and the impact of age on serum cTnI concentrations remains difficult to fully interpret.

Limitations of the present study largely relate to the small sample sizes, especially in subgroups of cats with cardiac disease. It has previously been proposed that ideally, 120 or more samples from healthy animals should be utilized to develop robust reference intervals [38]. However, given how difficult it can be to collect such a large number of samples from healthy individuals, others have suggested that a much smaller sample size can be sufficient [39]. Thus, the present sample size of 51 healthy cats may or may not be underpowered. Based on this study we propose a reference interval of <8 pg/mL- 190 pg/mL for healthy cats. Another limitation is the small sample size in cats with various forms of cardiac disease that likely contributed to the lack of significant differences across these groups (i.e., type II error). Lastly, due to small sample sizes, cats diagnosed with TMT based on elevated AC-cTnI-HS concentration in combination with supportive serial echocardiographic findings were grouped together with cats suspected to have myocarditis based on what the attending clinician deemed to be a severely elevated AC-cTnI-HS concentration. Given the role of the AC-cTnI-HS concentration in defining these cases prior to validation of the assay, the authors chose not to include these cats in the statistical analyses, and they were included for comparison purposes only.

## Conclusions

The AC-cTnI-HS assay is precise, reproducible, linear, and accurate for measurement of cTnI concentrations in serum from cats when assessed with pools ≥ 8 pg/mL. This study also supports the previously reported clinical utility as it was able to detect differences in serum cTnI concentrations between healthy cats and those with cardiac disease. The reference interval for serum cTnI concentration based on 51 healthy cats using this assay was determined to be < 8–190 pg/mL.

## Supporting information

S1 File Raw data associated with analytical validation of the AC-cTnI assay in cats including inter- and intra-assay variability, dilutional parallelism, and spiking recovery. (XLSM)

S2 File Raw data associated with clinical samples in healthy cats and cats with cardiac disease. (XLSX)

## References

[1] Langhorn R, Willesen JL. Cardiac Troponins in Dogs and Cats. J Vet Intern Med. 2016;30(1):36–50. doi: 10.1111/jvim.13801 26681537 PMC4913658

[2] Hanås S, Larsson A, Rydén J, Lilliehöök I, Häggström J, Tidholm A, et al. Cardiac troponin I in healthy Norwegian Forest Cat, Birman and domestic shorthair cats, and in cats with hypertrophic cardiomyopathy. J Feline Med Surg. 2022;24(10):e370–9. doi: 10.1177/1098612X221117115 36073987 PMC9511503

[3] Langhorn R, Willesen JL, Tarnow I, Kjelgaard-Hansen M, Koch J. Cardiac troponin I in three cat breeds with hypertrophic cardiomyopathy. Vet Rec. 2016;178(21):532. doi: 10.1136/vr.103549 27076529

[4] Langhorn R, Tarnow I, Willesen JL, Kjelgaard-Hansen M, Skovgaard IM, Koch J. Cardiac troponin I and T as prognostic markers in cats with hypertrophic cardiomyopathy. J Vet Intern Med. 2014;28(5):1485–91. doi: 10.1111/jvim.12407 25056593 PMC4895561

[5] Borgeat K, Sherwood K, Payne JR, Luis Fuentes V, Connolly DJ. Plasma cardiac troponin I concentration and cardiac death in cats with hypertrophic cardiomyopathy. J Vet Intern Med. 2014;28(6):1731–7. doi: 10.1111/jvim.12459 25319115 PMC4895638

[6] Herndon WE, Kittleson MD, Sanderson K, Drobatz KJ, Clifford CA, Gelzer A, et al. Cardiac troponin I in feline hypertrophic cardiomyopathy. J Vet Intern Med. 2002;16(5):558–64. doi: 10.1892/0891-6640(2002)016<0558:ctiifh>2.3.co;2 12322706

[7] Hori Y, Iguchi M, Heishima Y, Yamashita Y, Nakamura K, Hirakawa A, et al. Diagnostic utility of cardiac troponin I in cats with hypertrophic cardiomyopathy. J Vet Intern Med. 2018;32(3):922–9. doi: 10.1111/jvim.15131 29660794 PMC5980312

[8] Hertzsch S, Roos A, Wess G. Evaluation of a sensitive cardiac troponin I assay as a screening test for the diagnosis of hypertrophic cardiomyopathy in cats. J Vet Intern Med. 2019;33(3):1242–50. doi: 10.1111/jvim.15498 30990935 PMC6524108

[9] Stack JP, Fries RC, Kruckman L, Kadotani S, Wallace G. Galectin-3 as a novel biomarker in cats with hypertrophic cardiomyopathy. J Vet Cardiol. 2023;48:54–62. doi: 10.1016/j.jvc.2023.06.003 37480722

[10] Wells SM, Shofer FS, Walters PC, Stamoulis ME, Cole SG, Sleeper MM. Evaluation of blood cardiac troponin I concentrations obtained with a cage-side analyzer to differentiate cats with cardiac and noncardiac causes of dyspnea. J Am Vet Med Assoc. 2014;244(4):425–30. doi: 10.2460/javma.244.4.425 24479456

[11] Connolly DJ, Brodbelt DC, Copeland H, Collins S, Fuentes VL. Assessment of the diagnostic accuracy of circulating cardiac troponin I concentration to distinguish between cats with cardiac and non-cardiac causes of respiratory distress. J Vet Cardiol. 2009;11(2):71–8. doi: 10.1016/j.jvc.2009.09.002 19879824

[12] Novo Matos J, Pereira N, Glaus T, Wilkie L, Borgeat K, Loureiro J, et al. Transient Myocardial Thickening in Cats Associated with Heart Failure. J Vet Intern Med. 2018;32(1):48–56. doi: 10.1111/jvim.14897 29243322 PMC5787177

[13] Romito G, Elmi A, Guglielmini C, Poser H, Valente C, Castagna P, et al. Transient myocardial thickening: a retrospective analysis on etiological, clinical, laboratory, therapeutic, and outcome findings in 27 cats. J Vet Cardiol. 2023;50:51–62. doi: 10.1016/j.jvc.2023.09.001 37924558

[14] Romito G, Fracassi F, Cipone M. Transient myocardial thickening associated with acute myocardial injury and congestive heart failure in two Toxoplasma gondii-positive cats. JFMS Open Rep. 2022;8(2):20551169221131266. doi: 10.1177/20551169221131266 36339325 PMC9629561

[15] Wang Y, Seo J. Transient myocardial thickening after routine ovariohysterectomy in a 15-month-old Ragdoll cat. J Small Anim Pract. 2024;65(8):648–52. doi: 10.1111/jsap.13722 38444263

[16] Vollmar C, Mitropoulou A, Hassdenteufel E, Hildebrandt N, Schneider M. Arterial thromboembolism in a cat with transient myocardial thickening. J Vet Cardiol. 2024;52:14–8. doi: 10.1016/j.jvc.2024.01.002 38342049

[17] Lalor SM, Gunn-Moore DA, Cash R, Foot A, Reed N, Mellanby RJ. Serum Cardiac Troponin I concentrations in cats with anaemia - a preliminary, single-centre observational study. J Small Anim Pract. 2014;55(6):320–2. doi: 10.1111/jsap.12210 24645736

[18] Connolly DJ, Guitian J, Boswood A, Neiger R. Serum troponin I levels in hyperthyroid cats before and after treatment with radioactive iodine. J Feline Med Surg. 2005;7(5):289–300. doi: 10.1016/j.jfms.2005.01.002 16182184 PMC10822355

[19] Sangster JK, Panciera DL, Abbott JA, Zimmerman KC, Lantis AC. Cardiac biomarkers in hyperthyroid cats. J Vet Intern Med. 2014;28(2):465–72. doi: 10.1111/jvim.12259 24350989 PMC4857992

[20] Porciello F, Rishniw M, Herndon WE, Birettoni F, Antognoni MT, Simpson KW. Cardiac troponin I is elevated in dogs and cats with azotaemia renal failure and in dogs with non-cardiac systemic disease. Aust Vet J. 2008;86(10):390–4. doi: 10.1111/j.1751-0813.2008.00345.x 18826510

[21] Langhorn R, Jessen LR, Kloster AS, Jensen AP, Koch J. Cardiac troponin I in cats with compromised renal function. J Feline Med Surg. 2019;21(10):985–91. doi: 10.1177/1098612X18813427 31551016 PMC11132240

[22] Bijsmans ES, Jepson RE, Wheeler C, Syme HM, Elliott J. Plasma N-Terminal Probrain Natriuretic Peptide, Vascular Endothelial Growth Factor, and Cardiac Troponin I as Novel Biomarkers of Hypertensive Disease and Target Organ Damage in Cats. J Vet Intern Med. 2017;31(3):650–60. 28387019 10.1111/jvim.14655 PMC5435049

[23] Pugliese M, Napoli E, La Maestra R, Or ME, Bilgiç B, Previti A, et al. Cardiac Troponin I and Electrocardiographic Evaluation in Hospitalized Cats with Systemic Inflammatory Response Syndrome. Vet Sci. 2023;10(9):570. doi: 10.3390/vetsci10090570 37756092 PMC10538112

[24] Pelander L, Bach MBT, Ljungvall I, Willesen JL, Koch J, Dreimanis K, et al. Evaluation of cardiac troponin I as a predictor of death in critically ill cats. J Vet Intern Med. 2023;37(2):403–11. doi: 10.1111/jvim.16635 36708236 PMC10061183

[25] Oxford EM, Giacomazzi FB, Moïse NS, Santilli RA. Clinical and electrocardiographic presentations of transient trifascicular block in three cats. J Vet Cardiol. 2018;20(3):204–12. doi: 10.1016/j.jvc.2018.02.002 29572123

[26] Konishi K, Sakamoto M, Satake C, Isaka M, Okazaki S, Kono S, et al. Perioperative changes in cardiac biomarkers in juvenile cats during neutering. Front Vet Sci. 2022;9:1008765. doi: 10.3389/fvets.2022.1008765 36268044 PMC9577090

[27] Côté E, Zwicker LA, Anderson EL, Stryhn H, Yu J, Andersen E. Effects of dexmedetomidine and its reversal with atipamezole on echocardiographic measurements and circulating cardiac biomarker concentrations in normal cats. J Am Vet Med Assoc. 2022;260(8):1–9. doi: 10.2460/javma.21.06.0299 35175929

[28] Langhorn R, Willesen JL, Tarnow I, Kjelgaard-Hansen M. Evaluation of a high-sensitivity assay for measurement of canine and feline serum cardiac troponin I. Vet Clin Pathol. 2013;42(4):490–8. doi: 10.1111/vcp.12085 24131244

[29] Winter RL, Saunders AB, Gordon SG, Miller MW, Sykes KT, Suchodolski JS, et al. Analytical validation and clinical evaluation of a commercially available high-sensitivity immunoassay for the measurement of troponin I in humans for use in dogs. J Vet Cardiol. 2014;16(2):81–9. doi: 10.1016/j.jvc.2014.03.002 24834862

[30] Osredkar J, Fabjan T, Kumer K, Tršan J, Poljančič L, Košir M, et al. Comparison of ADVIA Centaur ultra-sensitive and high-sensitive assays for troponin I in serum. Pract Lab Med. 2022;31:e00293. doi: 10.1016/j.plabm.2022.e00293 35860388 PMC9289728

[31] Musetti V, Masotti S, Prontera C, Storti S, Ndreu R, Zucchelli GC, et al. Evaluation of the analytical performance of a new ADVIA immunoassay using the Centaur XPT platform system for the measurement of cardiac troponin I. Clin Chem Lab Med. 2018;56(9):e229–31. doi: 10.1515/cclm-2018-0054 29679526

[32] Clerico A, Zaninotto M, Padoan A, Masotti S, Musetti V, Prontera C, et al. Evaluation of analytical performance of immunoassay methods for cTnI and cTnT: From theory to practice. Adv Clin Chem. 2019;93:239–62. doi: 10.1016/bs.acc.2019.07.005 31655731

[33] Wesselowski S, Lidbury J, Saunders AB, Gordon SG, Suchodolski JS, Steiner JM. Analytical validation, sample stability, and clinical evaluation of a new high-sensitivity cardiac troponin I immunoassay for use in dogs, with comparison to a previous ultrasensitive assay. PLoS One. 2023;18(7):e0288801. doi: 10.1371/journal.pone.0288801 37463140 PMC10353792

[34] Luis Fuentes V, Abbott J, Chetboul V, Côté E, Fox PR, Häggström J, et al. ACVIM consensus statement guidelines for the classification, diagnosis, and management of cardiomyopathies in cats. J Vet Intern Med. 2020;34(3):1062–77. doi: 10.1111/jvim.15745 32243654 PMC7255676

[35] Welsh P, Preiss D, Shah ASV, McAllister D, Briggs A, Boachie C, et al. Comparison between High-Sensitivity Cardiac Troponin T and Cardiac Troponin I in a Large General Population Cohort. Clin Chem. 2018;64(11):1607–16. doi: 10.1373/clinchem.2018.292086 30126950 PMC6398571

[36] Lowry MTH, Doudesis D, Wereski R, Kimenai DM, Tuck C, Ferry AV. Influence of Age on the Diagnosis of Myocardial Infarction. Circulation. 2022;146(15):1135–48. 36106552 10.1161/CIRCULATIONAHA.122.059994 PMC9555758

[37] Serra M, Papakonstantinou S, Adamcova M, O’Brien PJ. Veterinary and toxicological applications for the detection of cardiac injury using cardiac troponin. Vet J. 2010;185(1):50–7. 20621713 10.1016/j.tvjl.2010.04.013

[38] Geffré A, Concordet D, Braun J-P, Trumel C. Reference Value Advisor: a new freeware set of macroinstructions to calculate reference intervals with Microsoft Excel. Vet Clin Pathol. 2011;40(1):107–12. doi: 10.1111/j.1939-165X.2011.00287.x 21366659

[39] Le Boedec K, Hugson N, McMichael M. Determination of Reference Intervals From Small Samples: How to Enhance Estimation Accuracy. Rotterdam, Netherlands: ECVIM-CA Congress; 2018.

